# Pancreatic cell fate specification: insights into developmental mechanisms and their application for lineage reprogramming

**DOI:** 10.1016/j.gde.2021.05.003

**Published:** 2021-05-29

**Authors:** Abigail Isaacson, Francesca M Spagnoli

**Affiliations:** Centre for Stem Cells and Regenerative Medicine, King’s College London, Guy’s Hospital, Floor 28, Tower Wing, Great Maze Pond, London SE1 9RT, UK

## Abstract

Diabetes is a group of metabolic disorders, which results from insufficient functional pancreatic β-cell mass either due to the autoimmune destruction of insulin producing β-cells, or their death or de-differentiation as compensation for insulin resistance. The ability to reprogram cell types within close developmental proximity to β-cells offers a strategy to replenish β-cell mass and a future possible treatment of diabetes. Here, we review recent advances in the fields of pancreas development and lineage reprogramming. We also probe the possibility of using reprogrammed cells as an approach by which to further understand developmental mechanisms, in particular roadblocks to changing cell identity. Finally, we highlight fundamental challenges that need to be overcome to advance lineage reprogramming for generating pancreatic cells.

## Introduction

**Figure 1:**
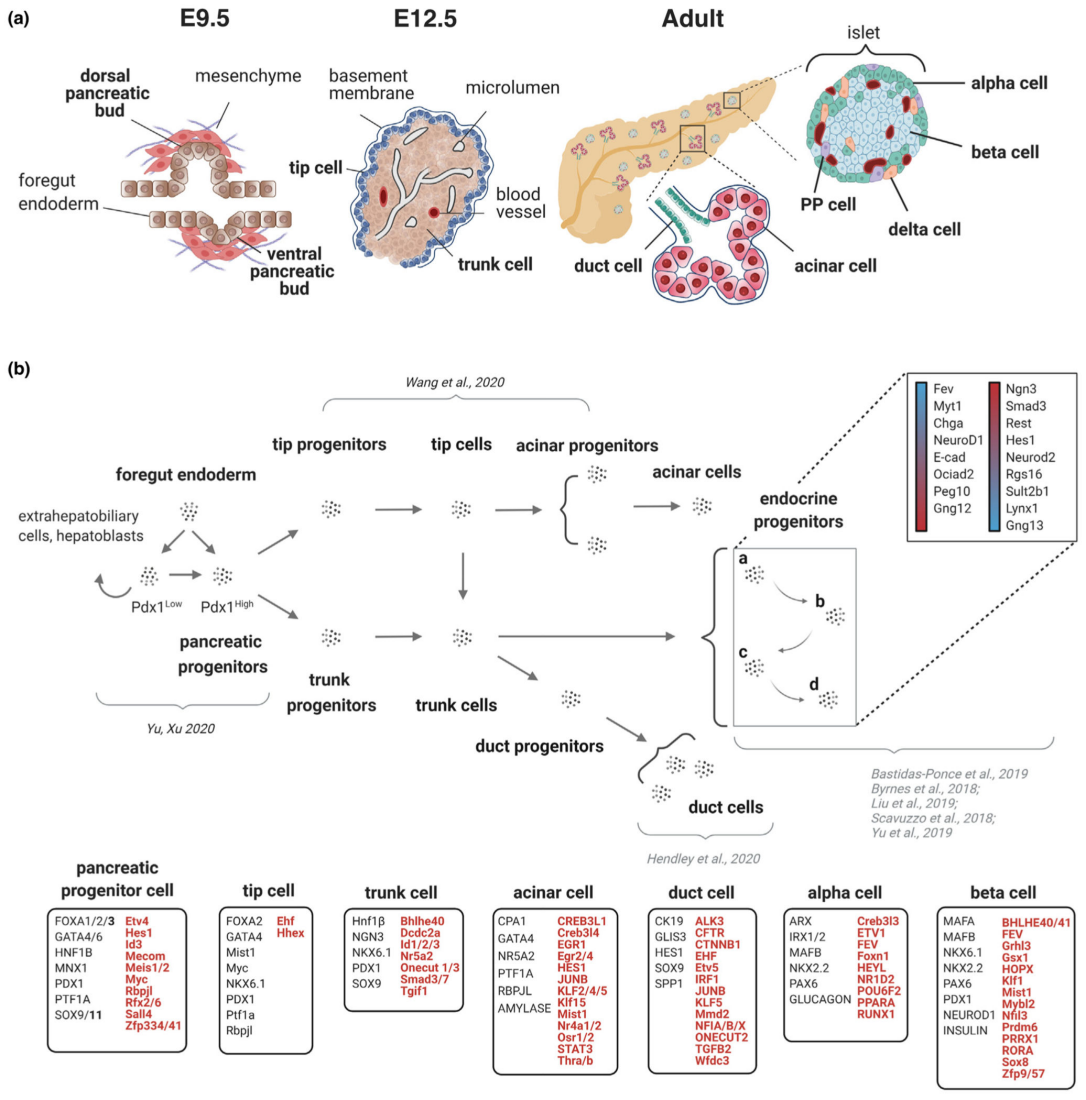
Model of pancreatic cell fate specification and differentiation. **(a)** Schematic representation of the developing pancreas at indicated developmental stages in the mouse embryo and after birth. PP, pancreatic polypeptide. **(b)** Lineage tree depicting key pancreatic cell subpopulations during embryonic development, constructed by incorporating recent findings from scRNA-seq studies of murine pancreatic tissue and predictions from computational models. Endocrine progenitors are shown in a box; labels a–d correspond to four identified transient populations. The bar colours ‘blue to red’ and ‘red to blue’ indicate whether the listed genes exhibit an overall increase or decrease, respectively, in expression from early (a) to late (d) endocrine progenitor states. Subset of TFs and marker genes typifying distinct stages and lineages are displayed in boxes; in red are genes identified in recent scRNA-seq studies [11,14,22,23••,^58^,^59^]. In capital are factors showing conserved expression pattern in human and mouse. Created with BioRender.com.

The pancreas is an endoderm-derived compound gland composed of multiple cell types that serve either its endocrine or exocrine functions [1]. In the exocrine pancreas, acini cluster around the terminus of each duct, which forms a tubular network that facilitates the transport of enzymes and zymogens synthesised by acinar cells (Figure 1). The endocrine pancreas, which represents the minority (1–2%) of the organ volume, consists of five hormone-producing cell types that are localised within the islets of Langerhans. The most abundant islet cell type is the insulin-secreting β-cell; these cells are responsible for maintaining glucose homeostasis by sensing blood glucose levels and secreting insulin accordingly into the islet vasculature [2]. Loss or dysfunction of β-cells results in diabetes, the most common pancreas-related disorder [3,4].

All pancreatic cell types arise from a common progenitor population that becomes specified as the result of temporal and spatial integration of extrinsic signals from the surrounding microenvironment and intrinsic genetic determinants. Numerous excellent reviews have been written on pancreatic development both in the mouse and human [1,2,5–7] and should be consulted for a more comprehensive view. Here, we summarize the main events of this multi-step process in Figure 1.

Despite the mapping of well-defined transcriptional networks and signaling pathways at key developmental junctions, our understanding of pancreatic cell fate specification is still ever-increasing; this is largely due to improvements in genetic cell isolation strategies used in combination with multi-omics tools that have enabled the identification of new genetic factors as well as subpopulations of progenitor cells and their progeny. Below, we discuss recent developments in the characterisation of distinct spatial and temporal molecular programmes responsible for mediating the specification of different pancreatic cell types. We also consider possible implications of recent findings in developmental biology to lineage reprogramming as well as the converse application of reprogramming to understand development. Indeed, successful experimental strategies for reprogramming of one specialized cell type into another typically rely on transcription factors (TF) that are associated with cell fate specification during embryonic development [8,9]. Particularly suited to initiate the cell fate change are a unique class of TFs, called pioneer factors, which can engage with silent chromatin, trigger its opening, and thus allow binding of other lineage-specific TFs [10]. Combinations of multiple TFs are often used to either facilitate a stepwise conversion from a progenitor to a mature state, for example with one TF being involved in fate specification and the others in subsequent differentiation, or alternatively one of the TFs might act as a repressor to erase the original cellular identity [8,9].

### Expanding the current view of the pancreatic transcriptional network

Pancreas development begins with the outgrowth of the foregut endoderm into a dorsal and ventral pancreatic bud at E9.0–E9.5 in mice and 26 dpc in humans [1,5,6]. Proliferation and specification of a population of multipotent pancreatic progenitors originating in both pancreatic buds occur largely due to the co-expression of key TFs, including Pdx1 and Ptf1a/p48, which, together with several other TFs, including Hnf1b, Foxa1/Foxa2 and Sox9, preserve their multipotency [2,5,7] (Figure 1).

The transcriptional landscape of pancreatic progenitors has further expanded thanks to many recent single-cell transcriptomic studies (reviewed in Ref. [11]; Figure 1b) and new lines of evidence have started shedding light on the mechanisms by which they function. A plethora of TF motif associations at the pancreatic progenitor stage has also been revealed using a combination of ATAC-seq together with TF motif enrichment analysis in mouse and human pluripotent stem cell (PSC)-derived pancreatic cells [12,13•,^14^,^15^]. Specifically, more than 50% of the sites that gained accessibility in hPSC-derived pancreatic progenitors contained FOXA motifs. FOXA transcriptional regulatory proteins are ‘pioneer factors’ that engage silent genes, helping to endow competence for cell-type specification [15]. Accordingly, genetic knockout studies of *FOXA2* in hPSCs revealed a requirement for *FOXA2* during human pancreatic specification for enhancer priming, which subsequently enables the recruitment of additional TFs [13•]. The analyses of the chromatin accessibility landscape will continue to elucidate developmental fate transitions and possibly uncover other pioneer factors or chromatin remodeling factors at play in pancreas specification.

In a similar vein, recent advances in single-cell technologies have led to the integration of complementary information about both cell lineage and cell state into synthesised views of differentiation dynamics [16]. An example of this is the integration of clonal history with single-cell RNA sequencing (scRNA-seq) by a ‘Cell Tagging’ approach [17,18••]. By labelling cells for the duration of a biological process, these approaches enable the construction of a developmental landscape complete with multi-level lineage relationships. scRNA-seq has also been used to dissect branching lineage trajectories of pancreatic cell types during development and for the identification of multiple transient cell populations (Figure 1b) [19,20]. Moreover, the possibility to detect transient populations of pancreatic cells and predict their terminal fates in the absence of clear temporal information has just recently been demonstrated [21••]. Here, each cell is assigned a probability of undergoing a fate change from a global map of computed fate potentials, so-called CellRank. By considering the ratio of spliced to unspliced mRNA transcripts (‘RNA velocity’), this approach enables the reconstruction of cell lineage trajectories as they arise during development and presents an improvement over earlier methods that have been limited to low-dimensional computations. Indeed, this method has led to the discovery of gene regulators likely to be driving the development of islet lineages for which very little is known, such as the somatostatin-producing δ-cells. Beyond development, CellRank has been applied to cells undergoing regeneration and could potentially be used to map the fate of single cells in other scenarios, including lineage reprogramming, where determining the direction of the process is often challenging.

In the developing pancreas, Neurogenin3 (Ngn3) is a transiently expressed TF necessary for the specification of all endocrine cell types [2,5]. Recent scRNA-seq analyses have helped to map how endocrine progenitors differentiate into endocrine subtypes and elucidate their differentiation trajectories [11,14,20,22,23••,^24^]. For instance, scRNA-seq of Ngn3^+^ cells using the Ngn3-Venus fusion reporter mouse line uncovered the differential expression of 58 genes that possess the same transient expression pattern as Ngn3 in the developing pancreas [22]. Additionally, expression of the TF Fev has been reported in several studies to define a distinct population of cells transitioning from endocrine progenitors towards terminally differentiated α-cells and β-cells [11,14,23••,^25^–^27^]. Byrnes *et al*. [23••] used pseudotime trajectory analysis and *in vivo* studies to characterize subpopulations, such as Fev^+^/Peg10^+^ cells and Fev^+^/Gng12^+^ cells, which are likely fated towards an α-cell and β-cell identity, respectively. Notably, a FEV^+^ population has also been identified in hPSC models of pancreas development [23••,^28^].

How Ngn3-expressing cells acquire specific endocrine cell fates has been also analysed with trajectory analysis of scRNA-seq data and combinatorial lineage tracing [29]. This study showed that co-expression of Ngn3 and Myt1 in endocrine progenitors biases them toward acquisition of β-cell fate. Moreover, Myt1^+^Neurog3^+^ cells displayed higher *Dnmt1* expression and enhancer methylation at the *Arx* gene locus, which encodes an α-cell fate promoting TF [29–31].

### Cell location and crosstalk with the microenvironment

The specification of different pancreatic cell types culminates in the formation of tip and trunk domains at E12 in mice (47 dpc in humans); at this stage, cells occupying the trunk domain are either duct or endocrine progenitor cells, while acinar progenitors are at the tip [5,7,28] (Figure 1b). The mechanisms that regulate this spatial patterning and the interdependence between architecture and cell differentiation are under investigation. Do the distinct pancreatic lineages derive from intrinsic predetermined progenitors? Or does fate specification depend on extrinsic signals from the surrounding niche? The answers to these questions are relevant for applications in cell replacement therapies for diabetes, including programming of PSCs and lineage reprogramming.

Recent studies suggest that a subset of predetermined unipotent progenitors exist even before organ patterning [32]. Clonal tracing has likewise been utilised to elucidate the lineage potential of precursor cells at ductal termini as well as the cellular dynamics leading to islet specification [33,34••]. Nyeng *et al*. [35] reconciled the opposing models of progenitor predetermination and niche instruction showing that differential cell-cell surface tension of progenitors determines cell sorting and initial pattern formation. Ultimately, the environmental signals in the trunk and tip niche(s) underpin cell fate determination [35]. Consistently, the same group showed that localised extracellular matrix (ECM) regulates the choice of bipotent progenitors in the trunk towards a ductal or endocrine fate [36••]. Here, enhanced Laminin deposition acts as an inducer of endocrine differentiation, while activation of Integrin α5 by Fibronectin triggers F-actin–YAP1–Notch signaling and inhibition of endocrinogenesis [36••].

There has been increasing interest lately in defining the composition of the microenvironment, which surrounds the embryonic pancreas, including the ECM and various cellular components. The mesenchyme is the most abundant cellular component of the microenvironment, exerting fundamental control on multiple aspects of pancreas formation [37]. Interestingly, sc-RNASeq has underscored a high degree of heterogeneity among mesenchymal cells in the developing pancreas, with 10 transcriptionally distinct subclusters being reported [23••]. Subpopulations of pancreatic mesenchymal cells have been also characterized *in vivo* using lineage tracing experiments and genetic studies in the mouse [38,39•]. These studies have provided insight into the requirement for organ-specific mesenchyme and unveiled heterogeneous mesenchymal cell populations equipped with a broad set of functions [38,39•]. Finally, deciphering cell-–cell interactions and ligand–receptor (L–R) pairs from transcriptomic datasets will help to elucidate how mesenchymal subpopulations provide specialized differentiation microenvironments and identify specialized *niches* underpinning pancreatic cell fate(s) specification. Learning how to recreate these niches with specific signaling molecules will help to instruct cells toward their ultimate fate during lineage reprogramming.

### Cellular plasticity and lineage reprogramming

**Figure 2:**
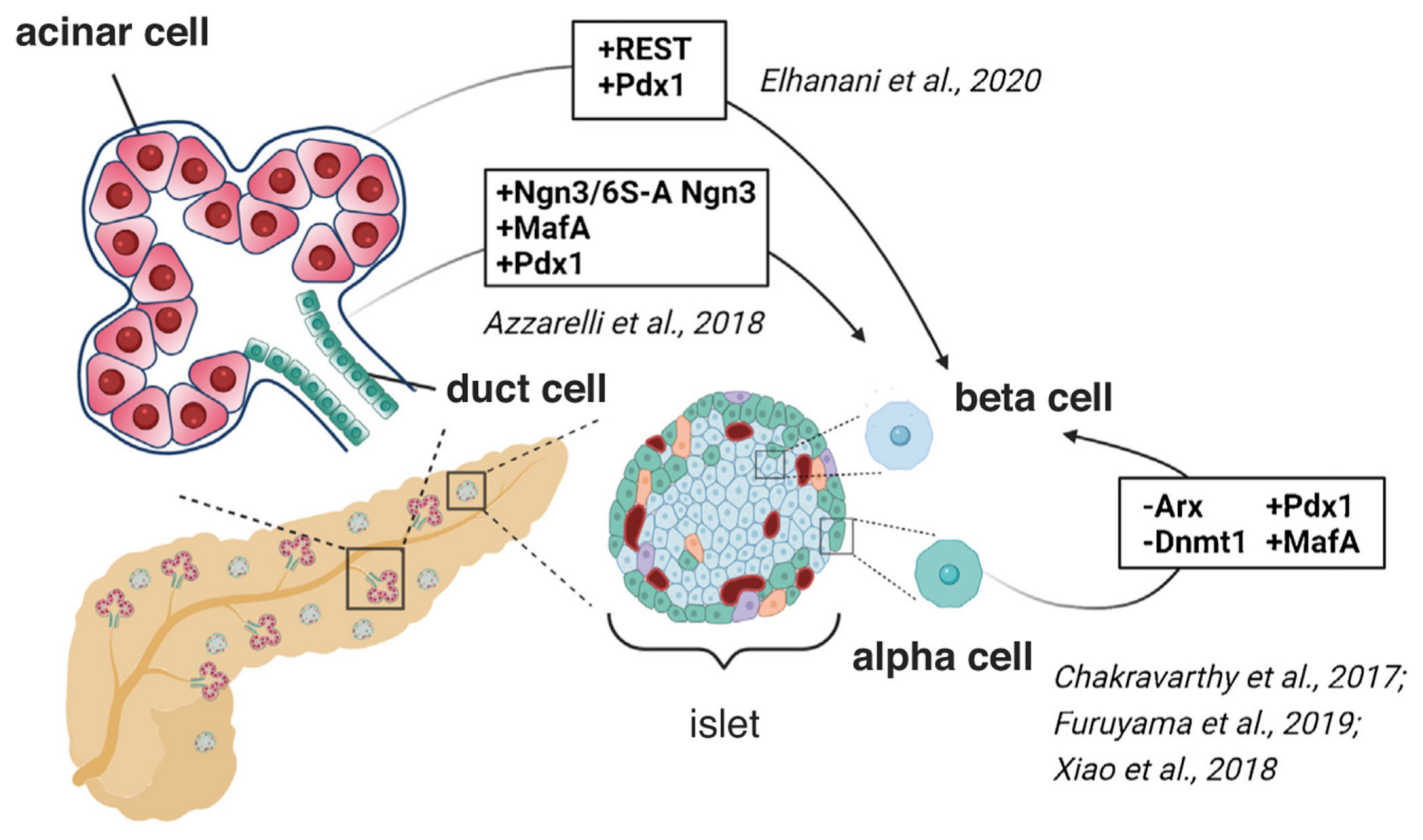
Visual overview of recently implemented reprogramming methods for pancreatic β-cell generation. Inhibition or upregulation of the relevant TFs are shown for each reprogramming route among adult pancreatic lineages. Created with BioRender.com.

A large body of evidence exists that points to a high degree of cellular plasticity in the adult pancreas [8,40]. It is now evident that differentiated pancreatic states, established during embryonic development, are reversible and cells can adopt another differentiated identity upon injury or under experimental conditions, using for instance cell type-specific TFs [8,41] (Figure 2). Seminal work from Melton and colleagues provided the first evidence that pancreatic acinar cells can be directed to acquire a β-cell identity upon *in vivo* forced expression of the TFs *Pdx1, MafA* and *Ngn3*, which play central roles in endocrine cell development and mature adult β-cells [42]. This has opened up doors to the exciting potential application of lineage reprogramming for generating therapeutically useful β-cell surrogates to cure diabetes.

Recent studies have highlighted α-cells as being highly suited for direct reprogramming into β-cells [41,43,44••,^45^,46••]. α-cells and β-cells share a common developmental origin, the same adult location in the islet and similarity in gene expression and chromatin modifications [47]. An ATAC-seq study has shown that the α-cell genome is remarkably accessible, supporting plasticity between these two islet cells [48]. Consistently, α-cells have been reported to convert into insulin-producing cells *in vivo* following extreme experimental (>99%) β-cell ablation in the mouse pancreas [45]. Signals intrinsic to the injured islets have been suggested to trigger the conversion of remaining α-cells into insulin expressing cells [49]. Further understanding of local signals that favour the fate switching in injury models will enhance β-cells regeneration through reprogramming of non-β-cells.

In the last few years, examples of TF-mediated lineage reprogramming of adult α-cells into insulin-producing cells, irrespective of β-cell loss, have been also reported. Using an elegant approach that combines lineage tracing and transgene delivery *via* pancreatic intraductal AAV infusion technique in the mouse, Xiao *et al*. [46••] showed that forced expression of Pdx1 and MafA (referred to as PM) induces *in vivo* α-to-β reprogramming. AAV-PM exposure proved partly effective also in streptozotocintreated human islets, in which insulin^+^ cells were induced after islet transplantation in diabetic mice [46••].

Ectopic expression of the same TFs has been further studied in individual islet cell types isolated from human primary islets by Herrera and colleagues [44••]. They showed that PM co-expression triggers efficient conversion of isolated human α-cells into insulin-producing cells, which acquired glucose-dependent insulin secretion and partial β-cell signature [44••]. Importantly, this study showed that human α-cells isolated from diabetic islets also preserve plasticity potential [44••]. Despite these very promising results in humans, clinical application and viability of such an approach may be hampered by the limited number of α-cells or other islet cells available for reprogramming and the accessibility to the pancreas. Another challenge in translating this approach is that both mouse and human PM-expressing α-cells obtained by lineage reprogramming display a hybrid transcriptome signature that is intermediate between α-cells and β-cells, not yet overlapping with native β-cells [44••,46••]. However, it is still unclear to which extent a reprogrammed β-like cell needs to erase its original identity to be fully functional and correct diabetes.

Relatively little is known about how lineage reprogramming is accomplished. A deeper understanding of the mechanisms underlying fate conversion between islet cell types may eventually yield molecular targets that allow the production of functional insulin-secreting cells at higher efficiency and closer to native islets. For instance, research into the genetic and epigenetic basis of α-cell identity revealed that combinatorial deletion of the TF Arx, which is required for α-cell fate specification, and Dnmt1, which is abundant in adult α-cells, significantly enhances reprogramming efficiency, leading to 50–80% *in vivo* conversion in the mouse [43]. These reprogrammed α-cells resembled native β-cells in multiple ways: at the transcriptome and electrophysiology levels and in their ability to secrete insulin in response to glucose [43]. Potential application of such a strategy to human islets remains to be tested.

Recent studies have started to shed light on mechanisms responsible for the reprogramming of other pancreatic cell types as well as non-pancreatic cells into β-cells. For instance, the transcriptional repressor REST has been identified as a regulator of PNM-mediated reprogramming in pancreatic acinar cells [50•]. Loss of *REST* combined with *Pdx1* expression results in the synergistic activation of endocrine genes [50•], which is consistent with previous observations in mouse endocrine progenitors [51]. Moreover, beyond the relevance for cellular reprogramming, this study has underscored a new REST-regulated activity in the development and maintenance of acinar cells [50•].

Another attractive approach to enhance reprogramming and β-cell generation is to exploit the post-translational regulation of reprogramming factors, such as Ngn3 [52,53]. The expression of a phospho-mutant Ngn3, which prevents phosphorylation of Ngn3 on CDK target sites and enhances protein stability, together with Pdx1 and MafA, improved the conversion efficiency of mouse pancreatic ductal organoids into insulin-expressing β-like cells [54]. A NGN3-based strategy has been also tested for reprogramming human ductal cells [55]. Understanding whether NGN3 post-translation regulatory mechanisms are conserved in humans could improve the reprogramming efficiency and generation of β-like cells starting from pancreatic duct organoids as source material.

### Closing remarks

The examples discussed here illustrate well how understanding the basic mechanisms underlying normal development canbeexploitedtodesignlineagereprogrammingstrategies. Conversely, studying lineage reprogramming is also a resource for understanding cellular plasticity within the pancreas as well as between the pancreas and other endodermal-derivatives (e.g. gut and liver), which represent alternative cellular sources for insulin-expressing cells [41,56,57].

While substantial progress has been made in dissecting pancreatic developmental programmes over the past few years, advances in lineage reprogramming lag behind. Future efforts should aim at further dissecting reprogramming processes at the single-cell level. Indeed, events or regulatory factors that are unique to the few reprogrammed cells are often masked in population studies. The availability of new approaches that can be performed at the single cell level on multiple cells at once, such as Tagging/Barcoding and Sequencing, will greatly improve the ability to study direct reprogramming dynamically [17,18••]. This will allow the unambiguous assessment of linear relationships between the initial and final identity acquired by the cells and define transitional states. Moreover, barriers to changing identity will be uncovered; these discoveries will possibly unfold new gatekeepers of cell identity as well as new reprogramming factors.

ScRNA-seq studies have defined additional TFs that might act as putative reprogramming factors and unveiled novel heterogeneity within the pancreatic islets, ductal tree and acinar tissues [58–60]. It is therefore plausible that populations within each pancreatic compartment might harbour distinct plasticity and reprogramming capacity. These new findings should be then integrated into novel reprogramming strategies. Finally, this is particularly important with regard to the new knowledge that is emerging of the pancreatic microenvironment [23••,^38^,39•]. Single cell-transcriptomics and computational predictions of L–R interactions will unveil niche factors and signaling cues that are at play *in vivo* during *β*-cell development. A more comprehensive understanding of these non-cell autonomous interactions may have significant implications for defining factors to integrate into reprogramming strategies. Thus, developmental studies and lineage reprogramming need to continue to go hand-in-hand to make progress toward the generation of cells for clinical applications.
